# Presence of TRPA1 Modifies CD4+/CD8+ T Lymphocyte Ratio and Activation

**DOI:** 10.3390/ph15010057

**Published:** 2022-01-01

**Authors:** Katalin Szabó, Ágnes Kemény, Noémi Balázs, Esam Khanfar, Zoltán Sándor, Ferenc Boldizsár, Rolland Gyulai, József Najbauer, Erika Pintér, Tímea Berki

**Affiliations:** 1 Department of Immunology and Biotechnology, University of Pécs Medical School, H-7624 Pécs, Hungary; balazs.noemi@pte.hu (N.B.); esam.khanfar@pte.hu (E.K.); boldizsar.ferenc@pte.hu (F.B.); najbauer.jozsef@pte.hu (J.N.); 2 Department of Pharmacology and Pharmacotherapy, University of Pécs Medical School, H-7624 Pécs, Hungary; kemeny.agnes@pte.hu (Á.K.); zoltan.sandor@aok.pte.hu (Z.S.); erika.pinter@aok.pte.hu (E.P.); 3 Department of Medical Biology, University of Pécs Medical School, H-7624 Pécs, Hungary; 4 Department of Dermatology, Venereology and Oncodermatology, University of Pécs Medical School, H-7624 Pécs, Hungary; gyulai.rolland@pte.hu

**Keywords:** TRPA1, lymphocytes, monocytes, CD4+ cells, CD8+ cells, B cells, TcR activation, imiquimod, intracellular Ca^2+^, cytokine, qRT-PCR, multiparameter flow cytometry

## Abstract

Transient Receptor Potential Ankyrin 1 (TRPA1) has been reported to influence neuroinflammation and lymphocyte function. We analysed the immune phenotype and activation characteristics of TRPA1-deficient mice (knockout—KO) generated by targeted deletion of the pore-loop domain of the ion channel. We compared TRPA1 mRNA and protein expression in monocyte and lymphocyte subpopulations isolated from primary and secondary lymphatic organs of wild type (WT) and KO mice. qRT-PCR and flow cytometric studies indicated a higher level of TRPA1 in monocytes than in lymphocytes, but both were orders of magnitude lower than in sensory neurons. We found lower CD4+/CD8+ thymocyte ratios, diminished CD4/CD8 rates, and B cell numbers in the KO mice. Early activation marker CD69 was lower in CD4+ T cells of KO, while the level of CD8+/CD25+ cells was higher. In vitro TcR-mediated activation did not result in significant differences in CD69 level between WT and KO splenocytes, but lower cytokine (IL-1β, IL-6, TNF-α, IL-17A, IL-22, and RANTES) secretion was observed in KO splenocytes. Basal intracellular Ca^2+^ level and TcR-induced Ca^2+^ signal in T lymphocytes did not differ significantly, but interestingly, imiquimod-induced Ca^2+^ level in KO thymocytes was higher. Our results support the role of TRPA1 in the regulation of activation, cytokine production, and T and B lymphocytes composition in mice.

## 1. Introduction

Emerging evidence indicates that TRPA1 is a molecular ionotropic receptor mediator [1] of the neuro-immuno-epithelial interface network [2,3,4] in the lung [5], skin [6] and gut [3,7,8] acting as a “pro-inflammatory hub” [9,10,11]. Absence, dysfunction or inhibition of TRPA1 has been shown to decrease inflammation- related symptoms of psoriasis [6,12,13], rheumatoid arthritis [14], actinic keratosis [15], atopic dermatitis [16,17,18], multiple sclerosis [19,20,21,22,23,24], and its function has been proposed to contribute to a variety of interrelated sensory and inflammatory processes such as inflammatory hyperalgesia [25,26], colitis [7,27], airway inflammation [28,29] and even oxidative stress storm syndromes in COVID-19 [30,31].

TRPA1 is a non-selective cation channel involved in sensation and sensitization to various exogenous stimuli such as cold, mechanical touch, and a plethora of inhaled, touched or orally consumed irritating cysteine-reactive agents and endogenous mediators of oxidative stress such as nitric oxide, hydrogen peroxide, and inflammatory signals [1,9,10,11,32,33]. Genetic disruption and pharmacological blockade of TRPA1 activity indicated that TRPA1 can be considered as part of a regulatory loop, an excitatory ion channel in primary sensory neurons in the peripheral nervous system, in keratinocytes and other non-neuronal cells, triggering the release of pro-inflammatory and inflammatory mediators such as calcitonin gene related peptide (CGRP), substance P (SP), IL-1β, IL-6 and IL-8 [34,35,36,37] Additionally, TRPA1 is responsive to a subset of inflammatory mediators such as bradykinins, histamines, eicosanoid, and prostaglandins [33,38,39] that activate inflammatory signaling pathways such as phosphoinositide-dependent phospholipase C (PLC-β) signaling with formation of inositol 1,4,5-triphosphate (IP3) and Ca^2+^ mobilization from intracellular stores and subsequent activation of either PI3K/NF-κB pathway or the ASK1/p38 pathway and subsequently CREB, or by cAMP signaling pathway through adenylate cyclase [40,41,42]. Furthermore, the TRPA1 function has been shown to be modulated by multiple factors, including Ca^2+,^ trace metals, pH, reactive oxygen species (ROS), nitrogen, and carbonyl species.

Its expression and functions has been described in various non-neuronal cells [1,43] such as keratinocytes [44], vascular smooth muscle cells [45], dendritic cells [46] in the gut [7,47], skin [48,49] and lungs [50], in peripheral blood leukocytes [14], in monocytes and macrophage-derived cell lines [51,52] and in lymphocytes, including CD4+ T cells [7,53]. However, both the level of expression and the role of TRPA1 in immune cells is still not unambiguously clear, primarily because of the controversial specificity of the applied antibodies [54], and the existence and regulatory effects of mouse and human TRPA1 splice variants [55,56]. Correlation between endogenous TRPA1 activity and T cell activation has been suggested by the effects of antagonists or gene-deficiency in pain-related in vivo treatments or by simultaneous changes observed in inflammation-related diseases [1,46,57].

The goal of this study was to reveal two potential aspects of TRPA1 function in lymphocytes: (1) its effects on the composition of lymphocytes in primary and secondary lymphatic organs in vivo; and (2) its role in TcR-mediated activation of lymphocytes in vitro. To answer these questions, we analyzed the immune phenotype of mononuclear cells using TRPA1-deficient mice generated by targeted deletion of the pore-loop domain of the ion channel and their activation characteristics.

Since we aimed to evaluate the role of TRPA1 in immune cells based on the comparison of wild type (WT) and the functional knockout (KO) TRPA1 mice, first of all we needed to know if any part of this tetramer-forming receptor was expressed in these mice. The engineered deletion eliminated the extracellular part of the pore-loop and a part of the sixth transmembrane domain [58,59,60]; this way the cation channel function of the receptor has been disabled [61], however the sensing of agonists is still present in the molecule if the truncated version is transcribed and translated, hence the receptor and signaling function of TRPA1 may still be active. Consequently, even low expression levels of the TRPA1 protein may modulate the function [62] and activation of immune cells.

## 2. Results

### 2.1. TRPA1 mRNA and Protein Expression in WT and Functional KO Mice

To test the influence of TRPA1 in a particular leukocyte function, first of all we needed to know which mononuclear immune cells express TRPA1 in our model system and whether the pore-loop truncated version of TRPA1 is present in KO mice cells. To evaluate the expression level of TRPA1 in mononuclear cells isolated from primary and secondary lymphatic organs, we analysed total RNA extracts of separated mononuclear cells of peripheral blood (PBMCs) and from spleen (splenocytes) and thymus compared to isolated trigeminus ganglions (TRG) by using qRT-PCR.

#### 2.1.1. Evaluation of TRPA1 mRNA Expression in Mononuclear Immune Cells of WT Animals

**Figure 1:**
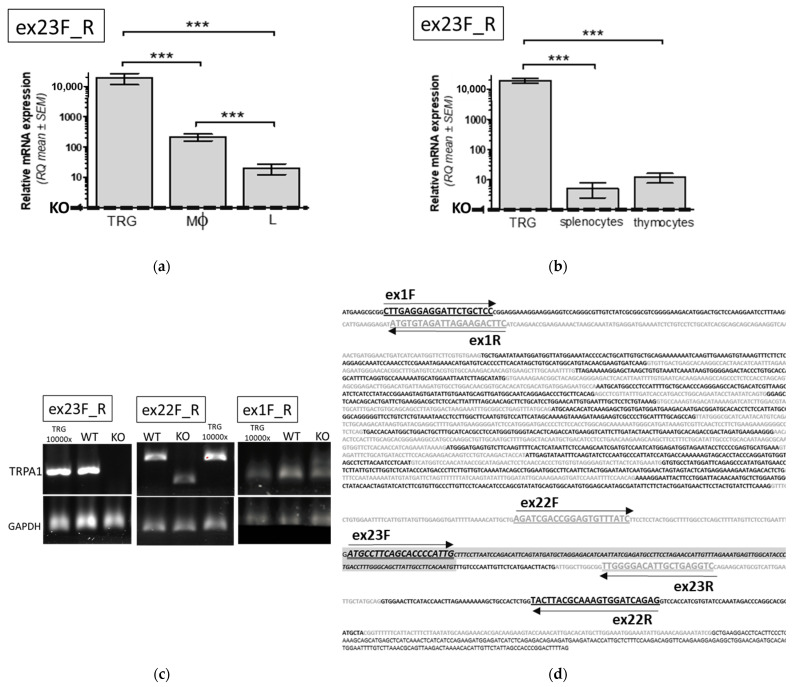
Quantitative RT-PCR analysis of TRPA1 mRNA expression in wild type mice related to the background of KO mice by using primers ex23F_R. Primer ex23F anneals to the deleted coding region of TRPA1 in KO mice, thus the relative mRNA levels (RQ) reflected the WT-specific transcripts compared to any potential non-specific background amplification in KO mice samples. RQ values are shown of (**a**) macrophage (Mϕ) and lymphocyte (L) cell fractions isolated from peripheral blood mononuclear cells or (**b**) peripheral mononuclear cells isolated from spleen or thymus as compared to RNA expression in trigeminal ganglion cells (TRG). Fold changes (RQ) were calculated according to 2-ΔΔCt methods, referenced to the non-specific background amplification in KO mice, comparing amplification from RNA samples (ΔC, normalized by GAPDH) of the WT mice and from RNA samples of the KO mice. Data are presented as mean ± SEM, (**a**) *n =* 4, (**b**) *n =* 6, *** *p* < 0.01. (**c**) Representative end point RT-PCR results by using three sets of primers depicted by arrows and names in (**d**). Mouse TRPA1 coding region with exon boundaries indicated by alternating black and grey type. Highlighted grey box indicates the deleted exon 23 region in KO mice that eliminates the pore-loop and most of the sixth transmembrane domain. Primer ex23F anneals to the deleted coding region, thus specific transcripts can only be detected in the WT immune cells by ex23F_R. By using primers ex22F_R resulted amplicons with different sizes in WT and KO cells. Primers ex1F_R used to amplify the first exon region generated a PCR product with RNA of TRG and Mϕ from TRPA1 WT or KO mice.

To evaluate which immune cells express TRPA1 mRNA in WT animals of our model system, first we used a set of primers specific to the deleted coding region in KO mice. This way even low level of mRNA expression (RQ) in WT cells relative to the background (“non-specific”) in KO mice could be calculated according to 2-ΔΔCt methods. As Figure 1a,b show, low but detectable levels of TRPA1 mRNA expression can be determined in monocytes/macrophage (Mϕ) and lymphocyte (L) fractions of peripheral blood, spleen (PMC) and thymus, though these expression levels were orders of magnitude lower than in sensory neurons of TRG. Figure 1a,b show relative expression level calculated as fold changes (RQ) as referenced by the non-specific background amplification in KO mice by using primers that span the deleted coding region. 

#### 2.1.2. Comparison of TRPA1 mRNA Expression in Immune Cells of WT and KO Mice

**Figure 2:**
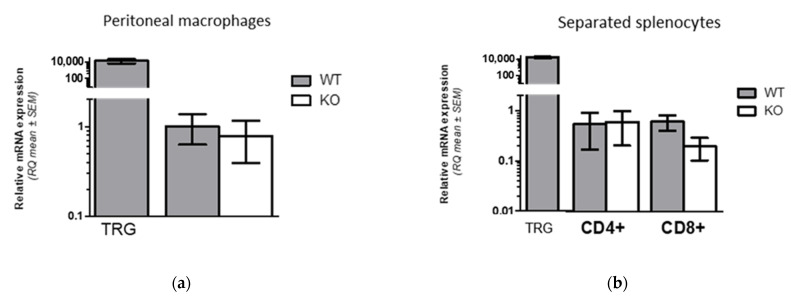

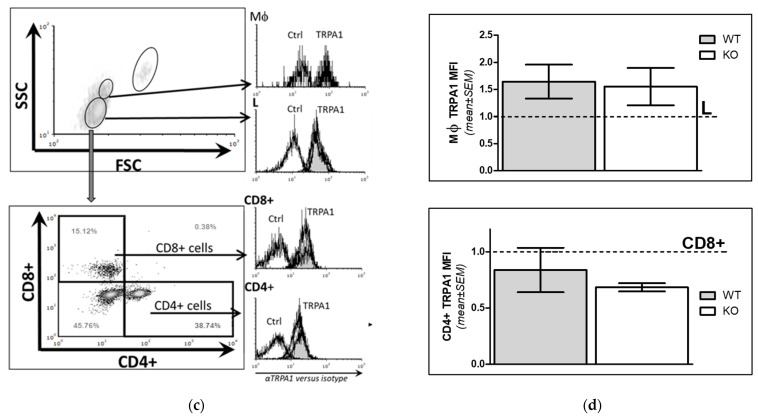
Analysis of TRPA1 expression in wild type and functional KO mice. (**a**,**b**) Quantitative RT-PCR analysis of relative mRNA (RQ) level and (**c**,**d**) TRPA1 protein expression. (**a**,**b**) Comparison of RQ of KO mice to that of wild type (WT) by using primers amplifying both wild type and truncated mRNA in KO mice (**a**) in peritoneal macrophages or (**b**) in separated CD4+ and CD8+ mononuclear cells isolated from spleen. Relative expression levels in these experiments were calculated as fold changes (RQ) referred to mRNA expression in monocyte/macrophage fractions of WT peritoneal cells. (**c**) Gating strategy and representative flow cytometry plots and histograms of peripheral blood cells stained by antibodies to CD4+, CD8+ and TRPA1 or isotype control (Ctrl) antibodies. (**d**) Comparison of TRPA1 protein expression of wild type (WT) to that of KO mice. Upper panel shows specific TRPA1 staining in macrophages relative to lymphocytes (dotted line -- L), lower panel shows that of CD4+ T cells relative to CD8+ cells (dotted line – CD8). Specific labelling of TRPA1 was calculated as mean fluorescent intensity (MFI) of Ctrl expression subtracted from MFI of TRPA1 staining. Data are presented as mean ± SEM, (**a**) *n =* 4, (**b**) *n =* 4, (**d**) *n =* 3. No significant differences in TRPA1 mRNA and protein expression were detected between samples from WT and KO cells by using primers amplifying both wild type and truncated mRNA and antibody recognizing the first 100 amino acids of the channel.

To evaluate whether the pore-loop truncated version of TRPA1 is present in KO mice cells, we characterized the mRNA level of WT and KO cells by using two further sets of primers: one that resulted in amplicons with different sizes in WT and KO cells and another that annealed to the first exon of TRPA1. Relative expression levels in these experiments were calculated as fold changes (RQ) referred to mRNA expression in monocyte/macrophage fractions. As Figure 2a demonstrates, TRPA1 transcripts were found in monocyte/macrophage fractions isolated from the peritoneal cavity in functional KO mice at a level comparable to that of WT. Similarly, as Figure 2b demonstrates, lower but detectable levels of TRPA1 mRNA expression were revealed in CD4+ and CD8+ T cells separated by fluorescence-based cell sorting of isolated spleen mononuclear cells of KO and WT mice.

#### 2.1.3. Comparison of TRPA1 Protein Expression Level in WT and KO Mice

Furthermore, comparison of intracellular immunofluorescence labeling of cells by an anti-TRPA1 antibody that recognizes the first 100 amino acids of the channel or labelling by its isotype control counterpart revealed that specific staining can be detected both in mononuclear cells of WT and KO mice (Figure 2c,d). This suggests endogenous TRPA1 protein expression in monocytes and CD4+, CD8+ lymphocytes both in WT and KO mice.

Altogether these results suggest low but detectable expression level of TRPA1 mRNA and protein in both WT and functional KO mice.

Our main question was whether this amount of functional TRPA1 channel present in WT lymphocytes did lead to detectable differences in development or in activation of T cells.

### 2.2. Phenotypic Analyses of WT and KO Lymphocytes

**Figure 3:**
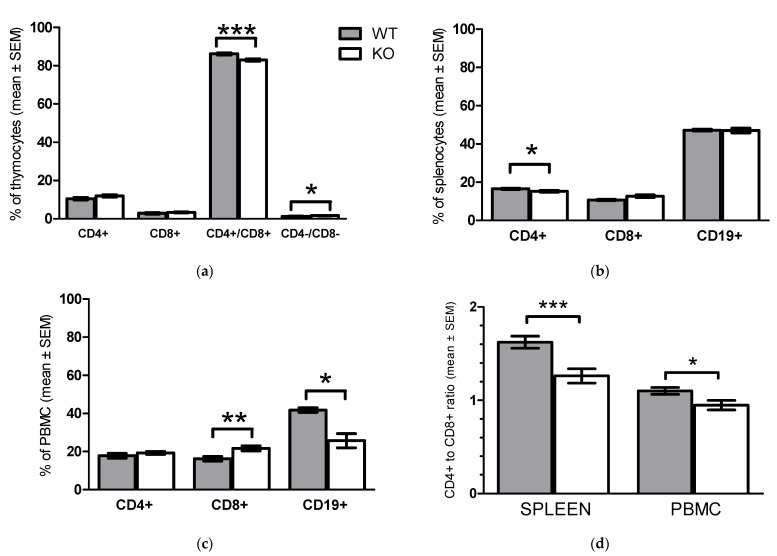
Phenotypic changes in functional KO mice. The percentage of CD4+, CD8+, CD4+/CD8+ or CD19+ mononuclear cells isolated from (**a**) thymus (**b**) spleen (**c**) peripheral blood is shown as comparison of wild type to functional KO mice. (**d**) Ratio of CD4+ T cells to CD8+ peripheral mononuclear cells purified from spleen or blood of WT and functional KO mice. Data are presented as mean ± SEM, (**a**) *n =* 7, (**b**) *n =* 4, (**c**) *n =* 3, (**d**) *n =* 8, * *p* < 0.05, ** *p* < 0.01, *** *p* < 0.001.

To answer this question, we compared the immune phenotype of primary and secondary lymphatic organs of functional KO and WT animals. In KO mice, significant decrease of CD4+/CD8+ DP thymocytes (Figure 3a), lower percentage of peripheral blood CD19+ B cells (Figure 3c), decreased ratio of CD4+ T cells in spleen (Figure 3b) and decreased CD4+/CD8+ T cell ratio in spleen and peripheral blood could be observed.

To study if presence of functional TRPA1 in lymphocyte subpopulation influences lymphocyte activation and immunological function, we characterized both early activation marker CD69, CD25 and TcR-mediated calcium signal in WT and KO animals.

### 2.3. Comparison of Early Activation Markers on CD4+ T Cells and CD8+ T Cells In Vivo in WT and KO Animals

**Figure 4:**
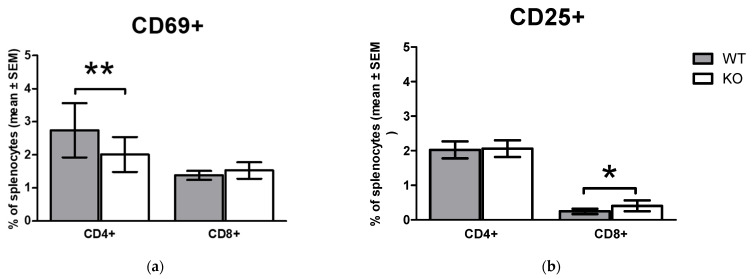
Expression of CD69 and CD25 activation markers on T cell subpopulations in vivo. (**a**) shows the ratio of splenic CD69+ (**b**) the ratio of CD25+ CD4+ and CD8+ T cells in wild type (WT) and functional KO mice. Data are presented as mean ± SEM, (**a**) *n =* 9, (**b**) *n =* 3, * *p* < 0.05, ** *p* < 0.01.

We also compared the expression of activation markers on the surface of WT and KO untreated T cells in vivo, which can provide further information on the role of functional TRPA1 on the in vivo activation of T lymphocytes. Significant differences were observed between the WT and KO mice in CD69 surface expression of splenic CD4+ T cells, an early activation marker by multiparametric flow cytometric analysis (Figure 4a). Reduced numbers of CD69+ cells were detected in CD4+ T cell population, while higher percentage of CD8+ T cells featured with surface appearance of CD25+ activation marker molecule in non-stimulated samples (Figure 4b).

### 2.4. Comparison of In Vitro TcR (CD3/CD28) Mediated Early Activation Marker CD69 Expression on T Cells of WT and KO Mice

**Figure 5:**
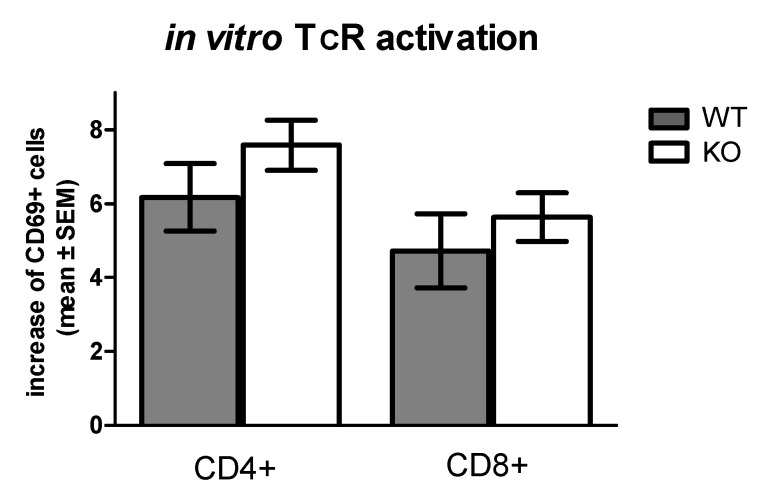
T cell receptor (TcR) stimulated expression of CD69 activation marker on T cell subpopulations in vitro. Elevation of CD69+ positive cells in CD4+ or CD8+ cell subpopulations is shown as the ratio of the percentage of CD69+ cells stimulated by anti-CD3/CD28 beads to that of unstimulated controls (CD69+ of stimulated/nonstimulated lymphocytes). Data are presented as mean ± SEM, *n =* 3, *p* > 0.05. No significant differences in activation marker CD69+ expression were detected between samples from WT and KO splenocytes.

While in non-stimulated lymphocytes in vivo expression of CD69 and CD25 were significantly different in WT and KO mice, we did not detect such difference in activated lymphocytes. As Figure 5 demonstrates, in vitro TcR stimulation by anti-CD3/CD28 beads induced 6.16 ± 0.91 fold increase of CD69+/CD4+ T cells in WT and 7.58 ± 0.68 fold increase in KO CD4+ T cells compared to quiescent non-stimulated cells. In CD8+ lymphocytes, TcR stimulation resulted 4.72 ± 1.00 fold and 5.63 ± 0.66 fold elevation of CD69 expression. The presence of the functional TRPA1 receptor did not modify significantly the elevation of CD69 expression in stimulated cells.

### 2.5. Comparison of In Vitro TcR and PMA/Ionomycin Stimulation-Induced Cytokine Release Profile of WT and KO Cells

**Figure 6:**
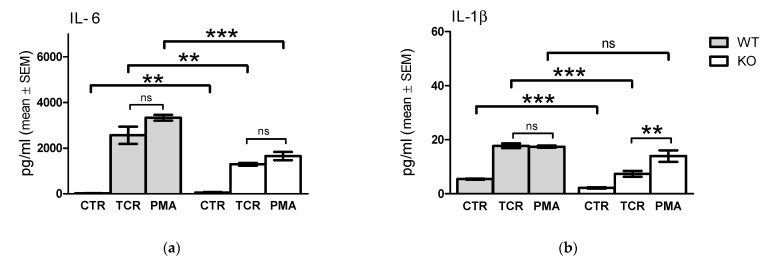

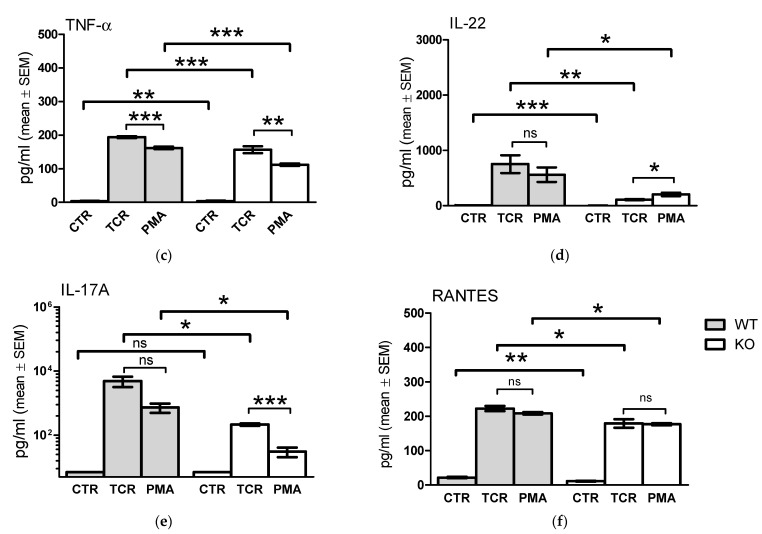
Comparison of TcR (via CD3/CD28) and PMA/ionomycin stimulated cytokine release of splenocytes from WT and KO animals. (**a**) Interleukin-6 (IL-6); (**b**) interleukin 1-beta (IL-1β); (**c**) tumor necrosis factor-alpha (TNF-α); (**d**) interleukin-22 (IL-22); (**e**) interleukin-17A (IL-17A); (**f**) Regulated on Activation, Normal T-cell Expressed and Secreted (RANTES) in lymphocyte cell cultures isolated from TRPA1 WT or KO animals following TcR or PMA treatments. Columns represent mean ± SEM. (**a**–**d**) *n =* 5, (**e**,**f**) *n =* 3, * *p* < 0.05, ** *p* < 0.01, *** *p* < 0.001, *ns* indicates non significant.

We found significant differences in inflammatory cytokine production between WT and KO derived lymphocyte cell cultures after TcR stimulation (Figure 6). The lack of functional TRPA1 receptor resulted in an overall reduced cytokine secretion from the TcR-stimulated KO cells relative to that of the WT cells, and a significantly decreased basal cytokine release by unstimulated KO cells, when compared to WT cells. Cytokine secretion stimulated by PMA/ionomycin treatment of WT or KO derived lymphocyte cultures were used as internal positive controls.

TcR stimulation induced significantly increased IL-6 expression in WT and KO cells (Figure 6a, WT control 16 ± 2 to TcR stimulated 2564 ± 378 versus KO control 41 ± 34 to TcR stimulated 1292 ± 60 pg/mL, respectively, mean ± SEM, *p <* 0.001, *n =* 6), but a more elevated cytokine secretion was induced by TcR stimulation of WT than KO cells (Figure 6a, *p* < 0.01). The absence of the functional TRPA1 caused a pronounced decrease in TcR stimulated secretion of IL-1β (Figure 6b, 18 ± 1 versus 7 ± 1 pg/mL, *p <* 0.01, *n =* 8), TNF-α (Figure 6c, 194 ± 3 versus 156 ± 11 pg/mL, respectively, mean ± SEM, *p <* 0.001, *n =* 8), IL-22 (Figure 6d, 753 ± 161 versus 111 ± 11 pg/mL, respectively, mean ± SEM, *p <* 0.01, *n =* 4), IL-17 (Figure 6e, 4940 ± 1176 versus 214 ± 18 pg/mL, respectively, mean ± SEM, *p <* 0.001, *n =* 4), and RANTES (Figure 6f, 223 ± 8 versus 179 ± 13 pg/mL, respectively, mean ± SEM, *p <* 0.05, *n =* 4) from KO cells.

By comparing TcR stimulated to PMA/ionomycin-induced cytokine release in WT cells, we observed significant differences only in TNF-α secretion (Figure 6c, 194 ± 2.7 versus 162 ± 3.9 pg/mL, respectively, mean ± SEM, *p <* 0.001, *n =* 8). Similarly, higher TcR stimulated TNF-α release was observed from KO splenocytes than as induced by PMA/ionomycin (156 ± 10.5 versus 112 ± 3.4 pg/mL, respectively, mean ± SEM, *p <* 0.01, *n =* 8). The levels of all secreted cytokines investigated were significantly lower in cultures of KO cells than of WT cells (*p <* 0.001). Both PMA treatment and TcR stimulation resulted in significantly increased IL-6 expression in WT and KO cells (Figure 6a), however a more elevated cytokine secretion was detected in WT cells than in KO splenocytes.

The absence of the functional TRPA1 resulted in lower TcR stimulated than PMA-stimulated secretion of IL-1β (Figure 6b) and IL-22 (Figure 6d) in KO cells, while in the cases of the other investigated cytokines secreted by WT cells, we did not detect significant differences between TcR- and PMA/ionomycin-stimulated cytokine release.

In contrast to TNF-α secretion, significantly lower levels of IL-1β (Figure 6b, 7.3 ± 1.08 versus 13.9 ± 2.1 pg/mL, respectively, mean ± SEM, *p <* 0.05, *n =* 8) and IL-22 (Figure 6d, 110.5 ± 10.8 versus 205.0 ± 32.3 pg/mL, respectively, mean ± SEM, *p <* 0.05, *n =* 4) were secreted after TcR stimulation than after PMA/ionomycin treatment of cultured KO cells. TcR stimulated IL-17 secretion was significantly higher than PMA stimulated from cells derived from KO mice, while only a higher tendency for IL-17 release by TcR stimulation was detected from WT mice derived cells (Figure 6e). No significant differences in RANTES secretion were detected in WT cells or KO cells (Figure 6f).

### 2.6. In Vitro TcR (anti-CD3) Stimulated Calcium Signal and Basal Intracellular Ca^2+^ Level in Lymphocytes and Thymocyte Subpopulations of WT and KO Mice

**Figure 7:**
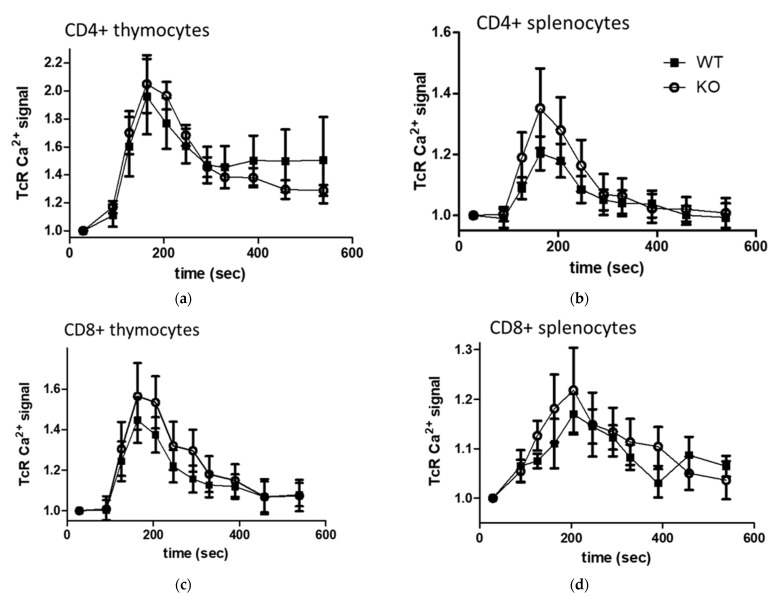
Comparison of calcium signals in thymocytes and splenocytes of TRPA1 WT and KO mice. (**a**–**d**) Isolated cells were labelled with anti-CD4 and anti-CD8 antibodies and then loaded with Fluo-3-AM. Fluo-3 fluorescence intensity that is proportional to the intracellular Ca^2+^ levels were detected by flow cytometry. Graphs show TcR stimulated time dependent changes in intracellular Ca^2+^ levels (**a**,**c**) in thymocytes and (**b**,**d**) in splenocytes of WT (squares) and KO (circles) animals. Activation of cells was induced by anti-CD3 cross-linking, fold changes in Ca^2+^- indicator fluorescence were calculated as ratio to that of quiescent cells in (**a**,**b**) anti-CD4+ or (**c**,**d**) anti-CD8+ labelled and gated subpopulations. Data are presented as mean ± SEM, (**a**,**c**) *n =* 6, (**b**,**d**) *n* = 3.

To assess the role of TRPA1 in TcR (anti-CD3 antibody) stimulated Ca^2+^ signal we compared the changes of free intracellular Ca^2+^ levels in thymocyte and splenocyte subpopulations of TRPA1 WT and KO mice by using flow cytometry. Significant differences in intracellular free Ca^2+^ levels of non-stimulated cells have only been detected between separated lymphocytes characterized by CD4, CD8 positivity but not between WT and KO animals. Likewise, we did not find significant differences between WT and KO animal cells in the CD3 induced (TcR induced) Ca^2+^ signal of neither cell types, or in the maximum response caused by ionomycin (2 μM) treatment applied at the end of each experiment as internal control. However, as Figure 7 shows, a tendency of higher Ca^2+^-peak was observed in CD4+, CD8+ T cells originated from KO (circles) compared to WT (squares) animals. Elevated activity of CD8+ splenocytes in TRPA1 deficient cells compared to TRPA1 +/+ lymphocytes has also been described [63] both in unstimulated and PMA/ionomycin stimulated cells.

### 2.7. Comparison of IMQ Induced Prolonged Ca^2+^ Signal in WT and KO Lymphocyte Subpopulations

**Figure 8:**
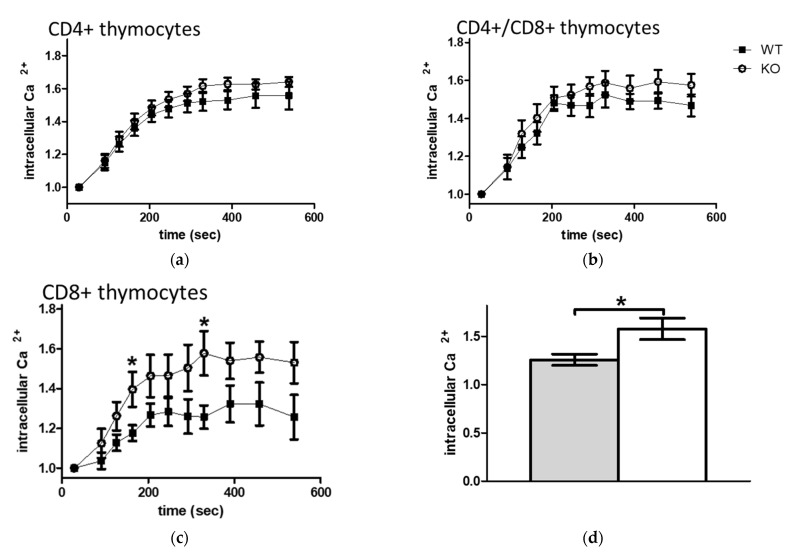
Analysis of IMQ stimulated changes of intracellular Ca^2+^ level in thymocytes of WT (squares and grey column) and KO (circles and open column) animals. Isolated cells were surface labeled with CD4 and CD8 antibodies and then loaded with Fluo-3-AM. Fluo-3 fluorescence that is proportional to the intracellular Ca^2+^ levels was detected by flow cytometry. Mean fluorescence intensity (MFI) of non-stimulated cells was measured, then imiquimod (100 µM) induced changes were monitored. Graphs show fold of changes in free intracellular Ca^2+^-levels calculated as ratio to that of quiescent cells in (**a**) CD4+ (**b**) CD4+/CD8+ double positive (DP), or (**c**,**d**) CD8+ thymocytes. Data are presented as mean ± SEM, *n =* 3, * *p* < 0.05.

Elevation of the free intracellular Ca^2+^ level itself has been reported to result from intracellular TRPA1 ion-channel gating [12,64,65,66], resulting in a sustained calcium signal. Imiquimod (IMQ), a frequently used inducing agent in modeling psoriasiform dermatitis, has been reported to elevate Ca^2+^ influx by both TLR7-dependent and independent mechanisms [67,68,69,70,71,72], and IMQ also has been reported to increase intracellular free calcium level by a TRPA1-dependent, but not Transient Receptor Potential Vanilloid-1 (TRPV1)-dependent mechanism [73]. To evaluate if in vitro stimulation by IMQ modulates intracellular Ca^2+^ levels of mononuclear immune cells, we monitored its time kinetics in thymocytes and splenocytes. As Figure 7 shows, a sustained elevation of intracellular free Ca^2+^ levels could be detected in all immune cell types treated with IMQ, though no significant differences have been observed in CD4+ and DP thymocytes and splenocytes (Figure 8a,b). However, IMQ induced a significantly higher level of intracellular Ca^2+^ in CD8+ thymocytes of KO mice compared to WT animals (Figure 8c,d).

## 3. Discussion

Here we provided evidence that expression of a pore-loop domain-deficient TRPA1 ion channel in mice (KO) results in changes in the immune phenotype when compared to WT counterparts. Namely, KO mice had lower double-positive thymocyte ratio, diminished CD4/CD8 rate in spleen and peripheral blood, and significantly lower B cell numbers in blood, when compared to their WT counterparts. Moreover, in vivo, the early activation marker CD69 level was diminished in CD4+ T cells of KO mice, while the CD25 level of CD8+ T cells was higher. Whether these differences were caused by some compensatory mechanisms that serves to recover the absence of TRPA1 function during development needs further evaluation. These results suggest that changes in TRPA1 channel activity may affect the development of immune cells in primary and secondary immune organs. Modulation of CD8+ T cells activity by TRPA1 function has also been described both in unstimulated and PMA/ionomycin stimulated spleen derived cells. An elevated higher oxygen consumption rate and extracellular acidification rate of TRPA1 deficient cells indicated that TRPA1 may play a role in the activity or development of CD8+ T cells [63] Absence of TRPA1 has been shown to profoundly influence melanoma progression, which may be related to a difference in immune surveillance of TRPA1 deficient mice [63]. Our results suggest that even a low level of functional TRPA1 ion channels is capable of influencing the composition of lymphocytes in vivo, compared to reshaping by a cation channel pore-deficient ionotropic receptor. To determine whether it is a direct impact of TRPA1 channel function in the particular immune cell subpopulation, or an indirect effect based on the distinct microenvironment of the innervated primary and secondary immune organs [74,75,76,77,78] by the effect of TRPA1 function, further studies will be needed. In addition to the existence of complex paracrine, autocrine and endocrine regulatory pathways between immune cells, neuronal cells and other non-neuronal cells in vivo in development of lymphocytes, there may be at least three potential intracellular mechanisms behind the observed changes in the immune phenotype of KO cells:

1. Functional TRPA1 monomers may form heterotetramers with other TRP channels (e.g., TRPV1)
2. Functional TRPA1 may modulate through changes in Ca^2+^ concentration in microdomains, and thereby:
  2(a) it may influence other Ca^2+^ sensitive plasma membrane Ca^2+^ influx channels in close proximity, such as P2X1 or P2X7 purinergic receptor channels, e.g., TRPV1 or other TRPs [79,80,81].
  2(b) it may influence signal transduction pathways through Ca^2+^ sensitive GPCRs e.g., A2B, adenylate cyclase, PLC β, or PIPK [10,46].

In neuronal cells, TRPA1 is generally co-expressed with TRPV1, and in vitro direct evidence was reported that formation of a channel by 2 molecules of TRPA1 together with two monomers of TRPV1 is possible [82], though in vivo has not been shown to form a heterotetramer channel. Physical interactions between members of different TRP channel subfamilies through formation of heteromultimeric complexes have been reported by numerous studies [83,84,85,86,87], sensitization of TRPV1 via activation of TRPA1 also has been described in vitro, which involves adenylyl cyclase, increased cAMP, subsequent translocation and activation of PKA, and phosphorylation of TRPV1 at PKA phosphorylation residues [88].

The absence of TRPA1 has been linked to the increased metabolic activity of CD8+ T cells [63], and has been shown to influence naive CD8+ T cell mitochondrial respiration and the glycolysis profiles of CD8+ T cells in TRPA1−/− and WT mice [63]. While we did not find significant differences in the CD3 induced (TcR induced) Ca^2+^ signals between WT and KO mice T cells, TRPA1 function modified the sustained elevation of intracellular calcium level of CD8+ T lymphocytes triggered by imiquimod. We aimed to show effects of agonists and antagonists in these cells, either by pre-incubation or by co-addition. These primary immune cells that were prepared from spleen and thymus of mice proved to be sensitive (vulnerable) to TRPA1 antagonists and agonists in our system, where flow cytometric analyses made it possible to follow cell shrinkage, granulation and membrane damage parallel with changes in the intracellular Ca^2+^ level. Unfortunately, at concentrations that have been used previously, successfully blocking mice TRPA1 function (50–100 μM of HC-030031, 0.5–1 μM A967079), cell shrinkage (measured by flow cytometry FSC values), granulation (measured by flow cytometry SSC values), and compromised membrane permeability (shown by PI positive staining of the cells) occurred, indicating necrotic cell death. Lower concentrations of antagonist (10–20 μM of HC, 0.1–0.2 μM A96) did not modified TcR dependent Ca^2+^ signal of the T cells. Addition of agonists or bimodal modulators, similarly, either had no effect on TcR dependent Ca^2+^ signal at lower concentrations or caused cell shrinkage and compromised membrane at higher concentrations in immune cells. Similar damage of these primary immune cells have been observed by these agents in the attempted cytokine release experiments. Inhibition by these agents on TRPA1 vary among species; a single amino acid mutation can affect the sensitivity of TRPA1, for example to HC-030031, and evaluation of immune cell type specific differences needs further investigations.

Our findings that specific protein staining was detected both in mononuclear cells of WT and KO mice suggested that at least the N-terminal part of the protein was expressed in monocytes, CD4+, CD8+ lymphocytes both in WT and KO mice. Our results also indicated the presence of a truncated transcript of TRPA1 in immune cells, similarly to that reported in Supplemental Data Figure S1 by Bautista et al. for TRG tissue of the same strain of TRPA1 functional deficient KO mice [58]. Expression of TRPA1 mRNA and protein in immune cells was reported based on comprehensive expression profile analysis of TRP channel gene families including TRPA1 in immune organs [89], end-point RT-PCR analysis, immunostaining by Western blot and other techniques or by functionality analysis in mouse immune cells, e.g., CD4+ splenocytes [7], in Th2 type T cells [44]. However, the levels of mRNA and protein expression have not been completely clarified by comparison to that of primary sensory neurons. Further analyses by using more sensitive methods for mRNA local expression, as single cell RNA scope analyses, and immunohistochemical analyses by monoclonal antibodies recognizing extracellular epitopes of TRPA1 channel may give more detailed answer of TRPA1 expression and localization in these immune cells.

We analyzed whether function of TRPA1 affects the TcR (CD3/CD28) induced secretion of pro-inflammatory cytokines as another inflammation-related activity of lymphocytes. Our results indicated significantly lower in vitro TcR induced secretion of numerous cytokines (IL-1β, IL-6, TNF-α, IL-17A, IL-22, and RANTES) from KO splenocytes than that of WT mice. Similarly, pro-inflammatory cytokine IL-6 expression has been reported significantly downregulated by TRPA1 deficiency in cartilage/chondrocytes [90], and another inflammatory mediator IL-8 has been shown to be upregulated in a TRPA1-dependent manner in human lung fibroblast cell lines [91].

In agreement with our results, some pro-inflammatory mediators, such as IL-1β, TNF-α, and IL-6 secretion stimulated by concanavalin A or anti-CD3/CD28 antibodies in vitro has also been shown to be decreased by TRPA1 antagonists [53] in isolated mouse splenocytes and human peripheral blood T cells. Bertin et al. described more sustained levels of calcium influx upon TcR stimulation and experienced distinct inflammatory profile in CD4+ T cells originated from IL-10−/−Trpa1−/− mice compared to wild-type TRPA1 harboring IL-10−/− cells [7]. This difference has been shown manifested in greater expression of the Th1 transcription factor Tbet and the Th1 cytokines IFN-γ and IL-2. Similarly, in human CD4+ T cells, TPRA1 knockdown increased IFN-γ and IL-2 production.

IL-22, IL-17A and RANTES (CCL5) are produced predominantly in Th1 cells during inflammatory processes [92,93]. IL-17 is a main effector cytokine in psoriasis that indirectly and directly induces keratinocyte proliferation and hyperplasia along with the secretion of inflammatory cytokines and chemokines forming a self-amplifying feed-forward response [94]. Dendritic and T cells in the dermis are known to generate a self-sustaining inflammatory loop along the TNF-α/IL-23/IL-17 axis [11]. The major inflammatory pathways, such as the NF-κB signal transduction pathway, promote the production of TNF-α, IL-1β, IL-6, and IL-12 p40 key inflammatory cytokines and also CCL2, CCL5, CXCL2, and CXCL10 chemokines [95,96,97]. Non-functional TRPA1 in the KO lymphocytes may dysregulate these pathways through influencing phosphoinositide-dependent phospholipase C (PLC β) signaling with subsequent formation of inositol 1,4,5-triphosphate (IP3) and Ca^2+^ mobilization from intracellular stores leading to the activation of the NF-κB pathway. Though TRPA1 proved not to be a key regulator of TcR (anti-CD3 only) stimulated calcium signaling in our studies, its function negatively modulated a sustained elevation of intracellular calcium level of CD8+ T lymphocytes triggered by imiquimod, the most frequently used in vivo psoriasis-inducing agent in model systems [87]. Our finding that in vitro treatment of lymphocytes by the synthetic purine imiquimod (IMQ) caused an elevated Ca^2+^ signal in TRPA1 KO CD8+ thymocytes raises another possible TRPA1-dependent mechanism through modulation of a purinergic receptor to trigger the cAMP signaling pathway through adenylate cyclase. It is still controversial how imiquimod triggers an increase in [Ca^2+^], but IMQ has been reported to act on purinergic receptors with the highest affinities to the A1, A2A and A2B [69,98]. IMQ also acts as a TLR7/TLR8 agonist and induces a subsequent expression of cytokines and stimulates antigen-presenting skin cells through adenosine receptors [98,99]. IMQ is able to induce intracellular Ca^2+^ increase mediated via TLR7 independent mechanisms, including the release of Ca^2+^ from internal stores and influx of the extracellular Ca^2+^. These mechanisms include activation of the PLC/IP3 pathway [100], opening the intracellular Ca^2+^ store through SERCA (sarco/endoplasmic reticulum Ca^2+^ ATPase) [101], and activation of TRPA1, but not the TRPV1 non-selective ion channel inducing Ca^2+^ influx as described by our group [73]. Neither macrophages nor microglia cells elicited calcium responses to IMQ [27], and considering that IP3Rs are found in all tissue types, the direct activation is not likely.

Our data add to the vast and growing field of neuroimmunology, where there is ample evidence of bidirectional communication between the nervous and immune systems, and support the role of TRPA1 as a ‘molecular hub’ involved in mediating such communication. The identification of receptors in immune cells that may recognize directly inhaled, touched or orally consumed cysteine-reactive irritating agents in lung, skin and gut will contribute to expand our understanding of the molecular and cellular basis of the neuro-immuno-epithelial interface network.

## 4. Materials and Methods 

### 4.1. Mice, Mononuclear Cell Isolation and Separation

Four to six week old male TRPA1 functionally deficient KO mice and their C57BL/6 WT counterparts [58,59] were used in groups of two to five in experiments. Trpa1+/+ and Trpa−/− lines were kept and bred in the facility of the Department of Pharmacology under conventional conditions; animals used in the experiment were genotyped by PCR analysis [102]. Spleens, thymuses and trigeminus ganglions (TRG) were isolated and homogenized mechanically, peritoneal macrophages were washed out from the peritoneal cavity, peripheral blood mononuclear cells (PBMCs) and spleen mononuclear cells (splenocytes) were separated either by hemolysis of samples or were isolated by Ficoll–Paque Plus (GE Healthcare, Chicago, IL, USA) density gradient centrifugation. Separation of CD4+, CD8+ or CD4+/CD8+ (DP) cells were based on fluorochrome-conjugated cell surface staining and sorting of cells by using antibodies anti-mouse CD4 Fluorescein (FITC, clone RM4-5, Cat. No 553046 BD Pharmingen, San Jose, CA, USA), and anti-mouse CD8α Phycoerythrin (PE, clone #53–6,7, Cat. No 553033, BD Pharmingen, San Jose, CA, USA) and Bio-Rad Cell Sorter S3E. For RNA isolation, monocytes/macrophage (M_ϕ_) and lymphocyte (L) fractions were isolated by culturing PBMC or peritoneal cells in RPMI supplemented with 10% fetal calf serum for 1 h, then attached and floating cells were separated by centrifugation. For RNA isolation, samples were dissolved in a TCEP supplemented buffer of NucleoSpin RNA XS kit (Macherey-Nagel) and stored at −80 °C.

### 4.2. Cell Stimulation, In Vitro Cell Culture 

For analyses of TcR- or PMA mediated cytokine release and appearance of surface activation marker CD69, 10^6^ isolated cells/sample were stimulated either by bead-conjugated anti-CD3/CD28 antibody (hereafter referred to as anti-CD3/CD28) as recommended in protocols of Dynabeads™ Mouse T-Activator CD3/CD28 for T-Cell Expansion and Activation kit (Cat. No 11453D, Thermo Fisher Scientific, Waltham, MA, USA), or a mixture with PMA/ionomycin [97], or were left unstimulated as controls in RPMI/5% FCS for 20 h at 37 °C. Supernatants were collected and stored at −20 °C until cytokine release analyses.

### 4.3. Cytokine Detection

To determine the concentrations of inflammatory cytokines/chemokines from cell culture supernatants, a Milliplex MAP assay based on the Luminex xMAP multiplexing technology was performed using a customized Milliplex Mouse Cytokine/Chemokine Magnetic Bead Panel (Merck KGaA, Darmstadt, Germany): 1. interleukin-1beta (IL-1β); 2. interleukin-6 (IL-6) 3. interleukin-17A (IL-17A); 4. tumor necrosis factor alpha (TNF-α); 5. interleukin-22 (IL-22); and Regulated on Activation, Normal T-cell Expressed and Secreted (RANTES/CCL5). Following previous optimizations, all samples were tested undiluted in a blind-fashion and in duplicates. The experiment was performed according to the manufacturer’s instructions. Briefly, 25 μL volume of each sample, control, and standard was added to a 96-well plate (provided with the kit) containing 25 μL of the capture antibody coated, fluorescent color-coded bead mixture. Biotinylated detection antibodies and streptavidin-PE were added to the plate after the appropriate washing and incubation periods. After the last washing step, beads were resuspended in 150 μL volume of drive fluid and was read in the Luminex MagPix instrument (MagPix 4.3.229.0, Luminex Corporation, Austin, TX, USA). Five-PL regression curves were generated to plot the standard curves for all analyte by the Belysa™ Immunoassay Curve Fitting Software (version 1.1.0, Merck KGaA, Darmstadt, German). Results are given in pg/mL.

### 4.4. RNA Isolation, cDNA Synthesis, and qPCR 

Total RNA was extracted from separated cells using the NucleoSpin RNA XS kit (Macherey-Nagel Inc., Bethlehem, PA, USA) according to the manufacturer’s instructions. Following cDNA generation (High Capacity cDNA Reverse Transcription Kit, 20, 6152 10 of 13 Thermo Fisher Scientific, Waltham, MA, USA), the TRPA1 mRNA expression was analyzed either individually (*n* = 3–5) or pooled (*n =* 5) of WT or KO mice samples using SensiFAST SYBR Lo-ROX Kit (Bioline, London, UK). Amplifications were performed using an Applied Biosystems 7500 RT-PCR System (Thermo Fisher Scientific, Waltham, MA, USA), analyzed with 7500 Software v2.0.6 (Thermo Fisher Scientific, Waltham, MA, USA) and normalized to GAPDH (a “housekeeping” gene) as reference. Fold changes (RQ) were calculated according to 2-ΔΔCt methods. Primer sequences were as follows: murine GAPDH forward: 5′-AATGGTGAAGGTCGGTGTG-3′, reverse, 5′-GTGGAGTCATACTGGAACATGTAG-3′; primers (ex1F, ex1R) annealing to the first exon of TRPA1 5′-CTTGAGGAGGATTCTGCTCC-3′ and 5′-GAAGTCTTCTAATCTACACAT-3′; primers (ex22F, ex22R) that resulted in amplicons with different sizes in WT (389bp) and KO (289 bp) cells: 5′-AGATCGACCGGAGTGTTTATC-3′ and 5′-CTCTGATCCACTTTGCG TAAGTA-3′, primers (ex23F, ex23R) span the deleted coding region specifically: 5′-ATGCCTTCAGCACCCCATTG-3′ and 5′-GACCTCAGCAATGTCCCCAA-3′ were also used for comparison of TRPA1 mRNA levels of various cell types in WT animals.

### 4.5. Flow Cytometry

Mononuclear cells were cell surface stained for phenotype analysis as described [103], followed by permeabilization and intracellular staining by anti-TRPA1 antibody (NOVUS NBP110-40763C) or rabbit IgG1 antibody as isotype control (NOVUS NBP2-36463C). For phenotyping of cells, we used appropriate combinations of the following antibodies purchased from BD Pharmingen, San Jose, USA): rat anti-mouse CD4 Fluorescein (FITC, clone RM4-5, Cat. No 553046), rat anti-mouse CD8a Phycoerythrin (PE, clone 53–6.7, Cat. No 553033), rat anti mouse CD8a PerCP (Clone 53–6.7, Cat. No 553036), hamster anti-mouse CD69 Fluorescein (FITC, clone H1.2F3, Cat. No 557392), rat anti-mouse CD4 Phycoerythrin (PE, clone H129.19, Cat. No 553653), rat anti-mouse CD8a PE-Cy5 (clone 53–6.7, Cat. No 553034), rat anti-mouse CD25 (clone 53–6.7 PC 61, Cat. No 557192). Monoclonal anti-mouse CD19-FITC (clone 1D3) was produced in our laboratory [104]. Briefly, after washing with phosphate-buffered saline containing 0.1% BSA (PBS/BSA), 10^6^ cells were labeled with fluorochrome-conjugated cell surface antibodies for 30 min in the dark. Cells were washed with PBS/BSA, then incubated in the presence of 4% PFA and 0.1% saponin containing fixation/permeabilization buffer for 20 min. Intracellular staining by anti-TRPA1 or isotype control antibodies were done in the presence of 0.1% saponin containing PBS/BSA for 30 min. Finally, cells were washed twice in PBS, then fixed with FACSFix (0.5% PFA in PBS, Sigma, Merck KGaA; Darmstadt, Germany) and measured on a FACSCalibur flow cytometer (Beckton Dickinson, Franklin Lakes, NJ, USA). Results were analyzed with FCS Express software (De Novo Software, Glendale, CA, USA).

### 4.6. Measurement of Intracellular Calcium Signaling

Intracellular free calcium was measured using Fluo-3AM (Molecular Probes, Cat. No. F-1242) according to the protocol described earlier [105,106,107]. Briefly, cells isolated and homogenized mechanically from the spleen or thymus were suspended in RPMI supplemented with 5% FBS, containing 2 mM CaCl_2_ (CaRPMI). Cells were stained with fluorochrome-conjugated cell surface antibodies, rat anti-mouse CD4 Phycoerythrin (PE, clone H129.19, Cat. No 553653), rat anti-mouse CD8a PE-Cy5 (clone 53–6.7, Cat. No 553034), then incubated with Fluo-3-AM (Molecular Probes Cat. No. F-1242) in a CO_2_ incubator for 30 min at 37 °C. Cell suspension was diluted in RPMI supplemented with 5% FBS and 2 mM CaCl_2_ (CaRPMI) [105], incubated for an additional 20 min, washed in CaRPMI and immediately measured in a Becton Dickinson FacsCalibur flow cytometer. Mean fluorescence intensity of Fluo-3 dye (FL1) was monitored in CD4+ or CD8+ subpopulations before and after stimulation with anti-mouse CD3 and crosslinking with anti-hamster IgG as described earlier [99,101] for 10 min. Activation of cells by imiquimod were done at 100 μM final concentration of IMQ (Tocris, Cat. No. 3700). Then ionomycin (2 μM, Sigma I 0634, Merck KGaA, Darmstadt, Germany) was applied at the end of every experiment for internal control to check nonresponsive cells and acquire a maximum response. Data were analysed by using the CellQuest program. Changes in Ca^2+^- indicator fluorescence were calculated as a ratio to that of non-stimulated quiescent cells.

### 4.7. Statistical Methods

Experiments were performed by comparison of at least three mice of each WT or KO genotype. Results were analyzed with GraphPad Prism 8.0 software (La Jolla, CA, USA). Data were expressed as mean ± SEM of the values received in independent experiments. Statistical evaluation was performed with SPSS v. 25.0 statistics package (IBM, Armonk, NY, USA) using Student’s *t*-tests and ANOVA, where *p*-values < 0.05 were considered significant.

## 5. Conclusions

Our results suggest that changes of TRPA1 activity may play a role in the development of immune cells in primary and secondary immune organs. Though TRPA1 proved not to be the key regulator of TcR- (CD3) stimulated calcium signaling, the lack of functional TRPA1 negatively modulated the TcR- (anti-CD3/CD28) induced inflammatory cytokine secretion of cells. Since a sustained difference in the elevation of intracellular calcium level of CD8+ thymocytes was triggered by imiquimod, activity of TRPA1 may play a role in sensitization to inflammatory processes, as in atopic dermatitis and inflammatory hyperalgesia, though uncovering the specific role of TRPA1 needs further investigation. Since the TRPA1 channel can be activated or modulated bimodally by a wide range of natural compounds [102,108], it raises the potential that these compounds may be considered for symptomatic treatment of these diseases by local regulation of TRPA1 activity in peripheral immune cells.

## Data Availability

Data is contained within the article.
